# Taxonomic Identity of the Invasive Fruit Fly Pest, *Bactrocera invadens*: Concordance in Morphometry and DNA Barcoding

**DOI:** 10.1371/journal.pone.0044862

**Published:** 2012-09-17

**Authors:** Fathiya M. Khamis, Daniel K. Masiga, Samira A. Mohamed, Daisy Salifu, Marc de Meyer, Sunday Ekesi

**Affiliations:** 1 African Fruit Fly Programme, International Centre of Insect Physiology and Ecology (icipe), Nairobi, Kenya; 2 Department of Biochemistry and Biotechnology, Kenyatta University, Nairobi, Kenya; 3 Molecular Biology and Biotechnology Department, International Centre of Insect Physiology and Ecology (icipe), Nairobi, Kenya; 4 Royal Museum for Central Africa, Tervuren, Belgium; University of Oxford, United Kingdom

## Abstract

In 2003, a new fruit fly pest species was recorded for the first time in Kenya and has subsequently been found in 28 countries across tropical Africa. The insect was described as *Bactrocera invadens*, due to its rapid invasion of the African continent. In this study, the morphometry and DNA Barcoding of different populations of *B. invadens* distributed across the species range of tropical Africa and a sample from the pest's putative aboriginal home of Sri Lanka was investigated. Morphometry using wing veins and tibia length was used to separate *B. invadens* populations from other closely related *Bactrocera* species. The Principal component analysis yielded 15 components which correspond to the 15 morphometric measurements. The first two principal axes contributed to 90.7% of the total variance and showed partial separation of these populations. Canonical discriminant analysis indicated that only the first five canonical variates were statistically significant. The first two canonical variates contributed a total of 80.9% of the total variance clustering *B. invadens* with other members of the *B. dorsalis* complex while distinctly separating *B. correcta*, *B. cucurbitae*, *B. oleae* and *B. zonata*. The largest Mahalanobis squared distance (D^2^ = 122.9) was found to be between *B. cucurbitae* and *B. zonata*, while the lowest was observed between *B. invadens* populations against *B. kandiensis* (8.1) and against *B. dorsalis s.s* (11.4). Evolutionary history inferred by the Neighbor-Joining method clustered the *Bactrocera* species populations into four clusters. First cluster consisted of the *B. dorsalis* complex (*B. invadens*, *B. kandiensis and B. dorsalis s. s.*), branching from the same node while the second group was paraphyletic clades of *B. correcta* and *B. zonata*. The last two are monophyletic clades, consisting of *B. cucurbitae* and *B. oleae*, respectively. Principal component analysis using the genetic distances confirmed the clustering inferred by the NJ tree.

## Introduction

Globally, Dacine fruit flies of the genus *Bactrocera* Macquart (Diptera: Tephrtitidae) are among the most important pests of fruits and vegetables [1]. In addition to the polyphagous nature of some species, several are considered highly invasive; aided by globalization of trade and poor quarantine infrastructure in the developing countries. Adults often exhibit a strong tendency for dispersal and the immature stages are readily transported to new areas via fruits movement [2]. In Africa, a member of the genus *Bactrocera* was detected in 2003 at the Kenyan coast [3] and later described as *Bactrocera invadens* Drew, Tsuruta & White [4]. The pest is believed to be native to Sri Lanka [5] and has rapidly expanded its geographical range, now reported from 28 African countries including the Indian Ocean archipelago of the Comoros [4], [6], [7], [8], [9], [10], [11], [12]. *Bactrocera invadens* is an emerging polyphagous fruit fly pest and in Africa it has been reported to attack over 43 fruit species from 23 families with mango being one of the most preferred cultivated host [10], [12], [13], [14]. Direct damage to mango due to *B. invadens* has been reported to range from 30–80% depending on the cultivar, locality and season [8], [12], [15]. In addition to the direct losses, indirect losses attributed to quarantine restrictions have been enormous. The direct and indirect damage continue to have wide reaching socio-economic implications for millions of rural and urban populations involved in the mango value chain across Africa. The pest has been described as “a devastating quarantine pest” by the Inter-African Phytosanitary Council [6].

Many economically important fruit fly pest species from the family Tephritidae belong to complexes of sibling species, presenting difficulties in identification, even to the experts [16]. *Bactrocera invadens* is believed to belong to the *B. dorsalis* (Hendel) complex of tropical fruit flies [4]. This complex comprises of more than 75 species largely endemic to South-East Asia [17] with undescribed species remaining in collections [18]. Indeed, the *B. dorsalis* complex of fruit fly species appear to be evolving rapidly demanding the need for closer assessment of their taxonomic identity through morphometric and genetic analysis. For example, Drew *et al.* [4] depicted different thoracic colourations of *B. invadens* that are morphotypes of the same pest but that has complicated the taxonomic identity of this pest.

Detail review of the *B. dorsalis* complex by Drew & Hancock [17] has led to considerable debate over species, and a number of published works has aimed at defining the limits of some species populations [19], [20], [21], [1]. A study by Tan *et al.* [22] compared the profiles of phenylpropanoid metabolites of four *Bactrocera* species from the *B. dorsalis* complex, that includes *B. dorsalis s.s.*, *B. invadens*, *B. correcta* and *B. zonata* and revealed that different profiles of phenylpropanoid ingredients in the rectal glands can be used for identification of these four species. Other studies on identification of the *B. dorsalis* complex by Schutze *et al.* [23] used geometric morphometric analysis of wing size and shape to discriminate species within this complex. However, recent observations by Drew *et al.* [5] emphasized the need to continue research on this complex to provide validity or otherwise, for all species in the complex, for both economic reasons and for refining the systematics of the Subfamily Dacinae. Due to the complexity of this group of fruit flies and the arrival of *B. invadens* into Africa, the need to undertake the inventory of the *B. dorsalis* complex in Asia and make comparison with what is in Africa becomes important in order to redefine this complex.

Morphometric analyses have been a useful technique in detecting morphological differences among organisms to distinguish closely related species including fruit flies, justify synonymies, demonstrate morphological variation along altitudinal or geographical gradients and propose new species [24], [25], [26], [27], [28], [29]. Indeed, in some frugivorous tephritid fruit fly species, diagnostic morphological characters for the identification of adult flies are now available [29], [30], [31], [5]. However, morphological tools present some limitations, mainly due to high homoplasy in most morphological characters and the existence of cryptic species groups across the family. Thus, the classification of Tephritids to the species level based on adult morphology alone is sometimes unreliable [32], [33], [34]. These limitations have led several taxonomists and quarantine officials alike to seek viable alternative ways of fruit fly identification including the use of molecular markers [35], [32], [36], [37]. Recently, the current molecular tool of choice is DNA Barcoding, which is a system that employs sequence diversity in short, standardized gene regions aiding in identification of species [38]. This standardized method for identifications of species focuses sequencing efforts on one target gene, cytochrome c oxidase subunit I (COI) [39], [40].

The main objective of our study was to establish whether *B. invadens* individuals collected from Africa could be distinguished from other Asian *Bactrocera* species using both multivariate morphometric analysis and molecular methods. Because *B. invadens* belongs to a complex, we believe that information generated from this investigation should help clarify its identity, resolve its placement in the right phylogeny, ease quarantine restrictions and potentially contribute to better management of the pest if sterile insect technique or eradication from particular locality or region becomes an option.

## Results

**Figure 1:**
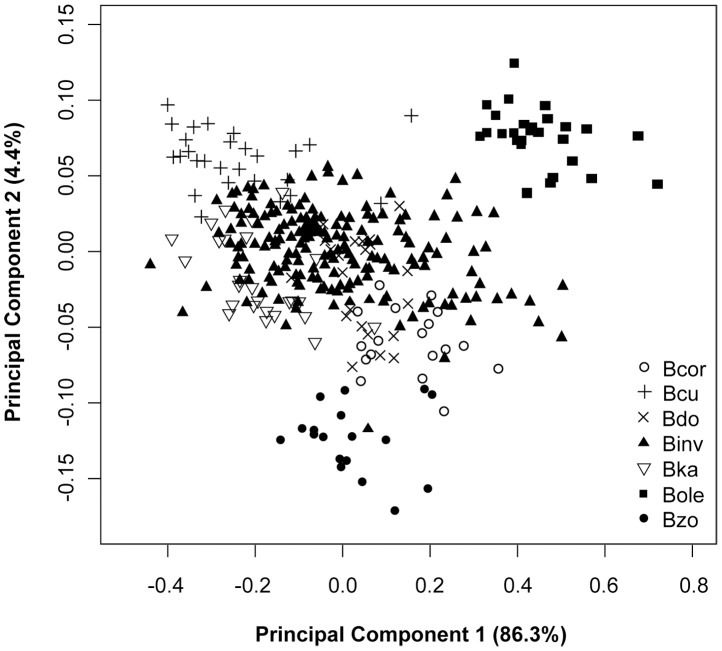
Projection of the wing and tibia data of *Bactrocera invadens* compared with the other *Bactrocera* species on the first two principal components.

The PCA yielded 15 components which correspond to the 15 morphometric measurements. Bartlett χ tests indicated that only the first 13 components were statistically significant. Projection of the morphometric data of the *Bactrocera* species on the first two principal axes showed a partial separation of these populations (Figure 1). The first two principal components contributed to 90.7% of the total variance (PC1 = 86.3% and PC2 = 4.4%) (Table 1). The third, fourth and fifth components contributed 2.3%, 2.0% and 1.7%, respectively, but did not improve separation of the populations. *Bactrocera invadens* populations and the other *Bactrocera* species belonging to the *B. dorsalis* complex could not be separated by PCA (Figure 1). However, the first two principal components separated *B. cucurbitae*, *B. oleae* and *B. zonata* into distinct groups (Figure 1). Contributions or loadings of the individual measurements indicate that PC1 represented the overall size, thus all loadings on PC1 are negative and within a small range (−0.34 to −0.15). PC2 is a contrast between vein 3, 4, 5, 6, 14 and tibia length with negative coefficients and the rest of the variables with positive coefficients hence PC2 is associated with wing shape (Table 1).

**Table 1:** Eigen values and coefficients (loadings) of the first two principal components (PC1 and PC2) for the log-transformed wing measurements data of the fruit fly populations.

	PC1	PC2
Proportion of variance	86.30%	4.40%
Eigen value	0.046 0.002	
Variable	Loadings	
Vein1(a_m)	−0.24	0.08
Vein2 (a_b)	−0.21	0.34
Vein3 (b_c)	−0.25	−0.05
Vein4 (d_e)	−0.34	−0.73
Vein5 (e_f)	−0.28	−0.21
Vein6 (f_g)	−0.31	−0.09
Vein7 (g_h)	−0.23	0.04
Vein8 (h_i)	−0.29	0.09
Vein9 (i_e)	−0.24	0.36
Vein10 (c_j)	−0.29	0.27
Vein11 (d_k)	−0.23	0.14
Vein12 (i_l)	−0.15	0.23
Vein13 (j_k)	−0.24	0.03
Vein14 (n_o)	−0.26	−0.01
Tibia length	−0.26	−0.04

**Figure 2:**
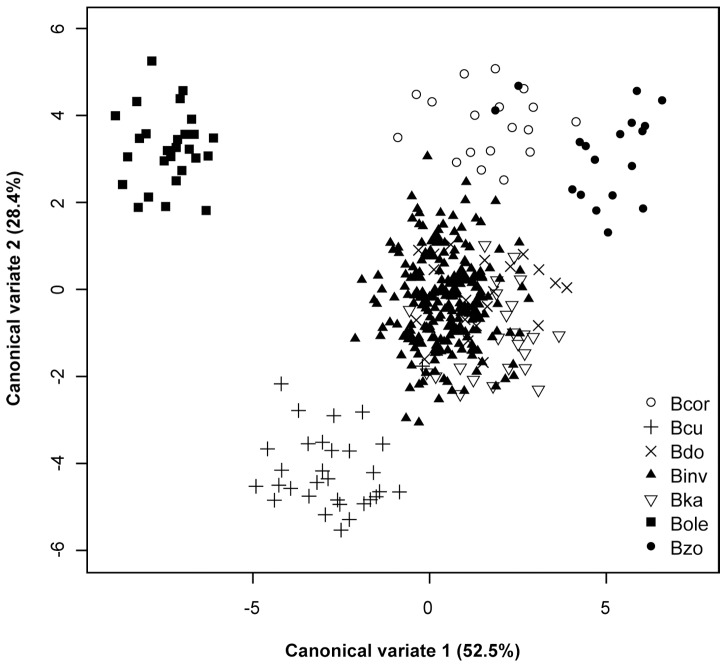
Projection of the wing and tibia data of *Bactrocera invadens* compared with other *Bactrocera* species on the first two canonical variates.

Tests for dimensionality for the canonical discriminant analysis revealed that only the first five canonical variates were statistically significant. Projection of the data on the first two canonical variate axes showed a better pattern of separation (Figure 2) compared with PCA. The first two canonical variates contributed a total of 80.9% (CV1 = 52.5% and CV2 = 28.4%) of the total variance. The third, fourth and fifth canonical variates contributed 9.3%, 5.7% and 2.9% of the total variance, respectively. The standardized canonical coefficients (Table 2) showed that CV1 is strongly dominated by positive correlation with vein4 and tibia length while strongly negatively correlated with vein1. CV2 is dominated by positive correlation with vein 14 and negative correlation with vein 3 and vein 10. *Bactrocera invadens* populations and the other *B. dorsalis* species complex clustered together while *B. correcta*, *B. cucurbitae*, *B. oleae* and *B. zonata* distinctly separated (Figure 2).

**Table 2:** Raw and standardized canonical coefficients for the canonical discriminant analysis on log-transformed wing measurements data for the fruit fly populations.

	Raw coefficients		Standardised coefficients	
Variable	CV1	CV2	CV1	CV2
Vein1(a_m)	−46.32	3.75	−1.71	0.14
Vein2 (a_b)	−16.96	−18.1	−0.63	−0.67
Vein3 (b_c)	14.87	−38.17	0.49	−1.26
Vein4 (d_e)	37.84	16.95	1.89	0.85
Vein5 (e_f)	−1.00	17.93	−0.04	0.78
Vein6 (f_g)	8.56	−19.66	0.39	−0.90
Vein7 (g_h)	17.82	4.65	0.68	0.18
Vein8 (h_i)	−12.93	−16.33	−0.59	−0.74
Vein9 (i_e)	2.50	18.49	0.11	0.85
Vein10 (c_j)	−2.01	−22.88	−0.10	−1.13
Vein11 (d_k)	−12.44	−22.2	−0.46	−0.82
Vein12 (i_l)	−22.96	27.55	−0.72	0.87
Vein13 (j_k)	−2.47	−6.96	−0.11	−0.32
Vein14 (n_o)	0.26	26.42	0.01	1.14
Tibia length	26.65	8.56	1.11	0.36

Mahalanobis distances used to compare morphometric divergence among populations group centroids showed a large degree of segregation in populations outside the *B. dosrsalis* complex and little interpopulation variability within the complex. For example, the largest Mahalanobis squared distance (D^2^ = 122.9) was found to be between *B. cucurbitae* and *B. zonata*, followed by *B. oleae* and *B. zonata* (111.8), *B. correcta* and *B. curcubitae* (88.4), and *B. cucurbiate* and *B. oleae* (68) (Table 3). Comparison of *B. invadens* populations against *B. dorsalis* sample gave a square distance of 11.4. The smallest distance was between the *B. invadens* populations and *B. kandiensis* (8.1).

**Table 3:** Mahalanobis Squared Distances (D^2^) between clusters representing the species/populations of *Bactrocera invadens* and other *Bactrocera* species.

Species	Bcor	Bcu	Bdo	Binvadens	Bka	Bole	Bzo
Bcor	-						
Bcu	88.4	-					
Bdo	21.8	48.4	-				
Binvadens	22.1	40.4	11.4	-			
Bka	26.5	43.1	15.9	8.1	-		
Bole	54.6	68.0	61.4	45.1	71.7	-	
Bzo	26.6	122.9	39.6	43.4	36.6	111.8	-

Bcor – *B. correcta*, Bcu – *B. cucurbitae*, Bdo – *B. dorsalis*, Binvadens – *B. invadens*, Bka – *B. kandiensis*, Bole – *B. oleae* and Bzo – *B. zonata*.

**Figure 3:**
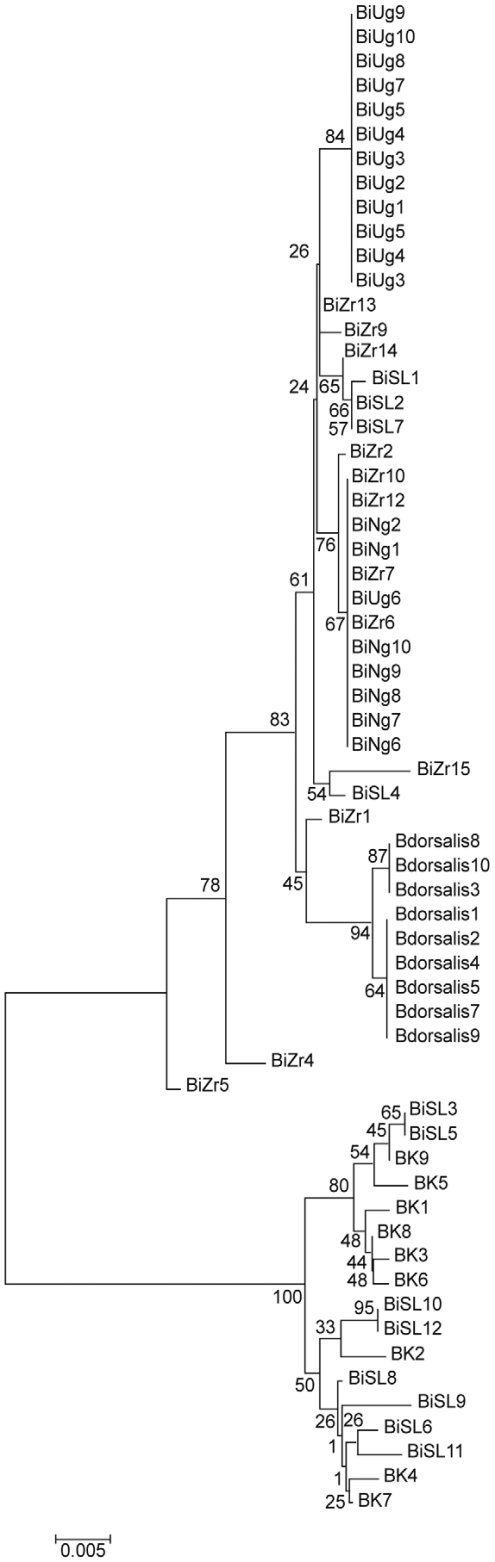
Evolutionary relationships between *Bactocera invadens* populations, *B. dorsalis s.s* and *B. kandiensis* as inferred using Neighbour-Joining method by Mega 5 program (Tamura *et al.*, 2011).

The first phylogenetic tree was derived considering only the species belonging to the *Bactrocera dorsalis* complex, the *B. invadens* populations from Kenya, Uganda, Zaria and Sri Lanka, *B. dorsalis s.s.* and *B. kandiensis*. The optimal tree with the sum of branch length = 0.14854468 is as shown in Figure 3. The percentage of replicate trees in which the associated taxa clustered together in the bootstrap test (1000 replicates) are shown next to the branches [41]. The tree is drawn to scale, with branch lengths in the same units as those of the evolutionary distances used to infer the phylogenetic tree. The evolutionary distances were computed using the Kimura 2-parameter method [42] and are in the units of the number of base substitutions per site. The analysis involved 62 nucleotide sequences. Codon positions included were 1st+2nd+3rd+Noncoding. All positions containing gaps and missing data were eliminated from the dataset (Complete deletion option). There were a total of 658 positions in the final dataset. The tree separated the *B. invadens* populations into two clusters (Figure 3). One cluster consisted of *B. invadens* populations from Kenya, Uganda, Zaria and Sri Lanka with a separate branch consisting of *B. dorsalis* population. The second cluster was dominated by *B. invadens* individuals from Sri Lanka that were grouped with *B. kandiensis* (Figure 3).

**Figure 4:**
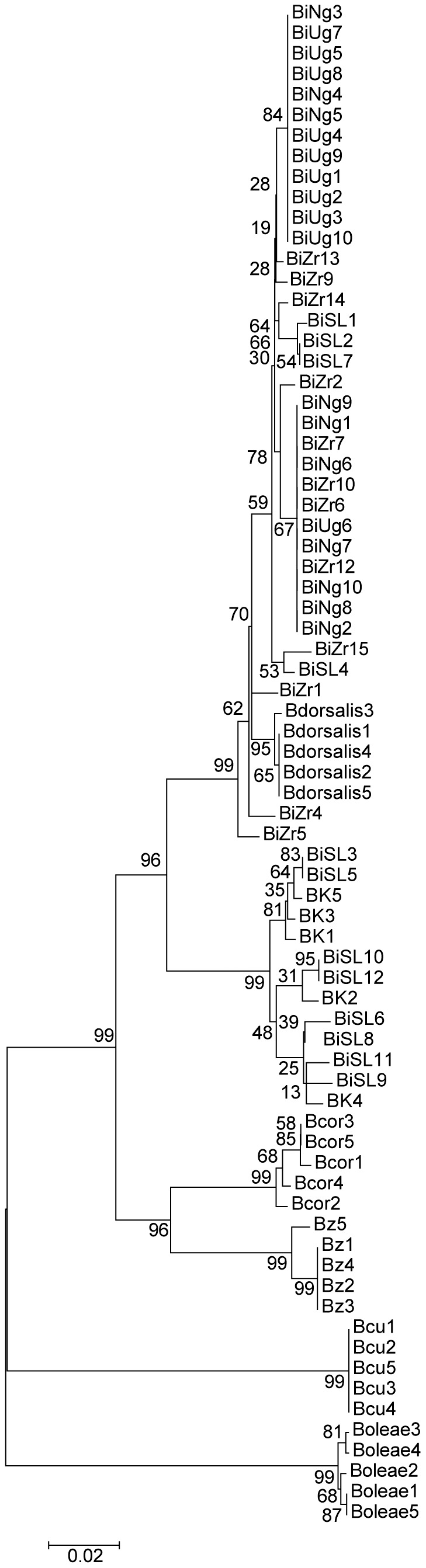
Evolutionary relationships between *B. invadens* populations and other *Bactrocera* species included in the study as inferred using Neighbour-Joining method by Mega 5 program (Tamura *et al.*, 2011).

The second tree was constructed for all the *Bactrocera* species for which DNA barcodes have been generated in this study. The optimal tree with the sum of branch length = 0.49981941 is shown in Figure 4. The percentage of replicate trees in which the associated taxa clustered together in the bootstrap test (1000 replicates) are shown next to the branches [41]. The tree is drawn to scale, with branch lengths in the same units as those of the evolutionary distances used to infer the phylogenetic tree. The evolutionary distances were computed using the Kimura 2-parameter method [42] and are in the units of the number of base substitutions per site. The analysis involved 74 nucleotide sequences. Codon positions included were 1st+2nd+3rd+Noncoding. All positions containing gaps and missing data were eliminated. There were a total of 658 positions in the final dataset. This analysis clustered the *Bactrocera* species populations into four groups (Figure 4). The first group had the clustering of the *Bactrocera dorsalis* species complex (*B. invadens*, *B. kandiensis* and *B. dorsalis sensu stricto*), branching from the same node. The second group consisted of *B. correcta* and *B. zonata* branching from the same node. While the last two groups are clades, consisting of *B. cucurbitae* and *B. oleae*, respectively (Figure 4).

**Figure 5:**
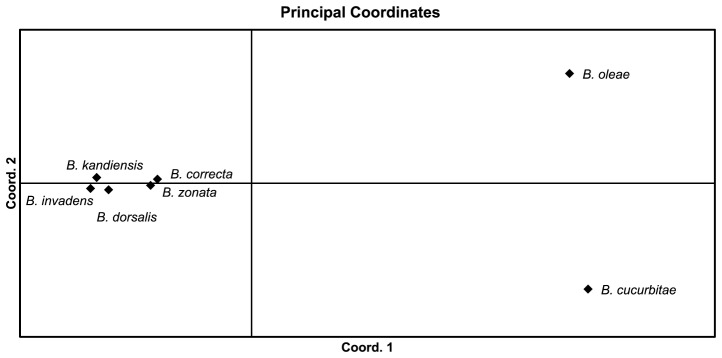
Plots of the principal coordinate analysis (PCA) from the covariance matrix with data standardization calculated using GenAlEx for the *Bactrocera* species.

The table of genetic distances (Table 4) was constructed by Mega 5 [43] using the Kimura 2-parameter model. The output was used to generate principal component plots, using GenAlEx 6.41 [44]. In this analysis of the *Bactrocera* species using principal coordinate analysis (PCA), the first two axes explained 59.38% of the variation (the first axis 34.93%, and the second axis 24.46%) (Figure 5). The PCA separated the seven species into four distinct clusters. A cluster was occupied by the species belonging to the *B. dorsalis* complex, a cluster consisting of *B. correcta* and *B. zonata*, *B. cucurbiate* on its own cluster and likewise, *B. oleae* (Figure 5).

**Table 4:** Estimates of Evolutionary Divergence over Sequence Pairs between Groups generated by Mega 5 program (Tamura *et al.*, 2011).

Species	Bcu	Bcor	Bzo	Binvadens	Bka	Bdo	Bole
Bcu	-						
Bcor	0.181	-					
Bzo	0.183	0.076	-				
Binvadens	0.176	0.098	0.105	-			
Bka	0.187	0.099	0.101	0.057	-		
Bdo	0.167	0.091	0.100	0.025	0.06	-	
Bole	0.194	0.175	0.185	0.177	0.175	0.17	-

Bcor – *B. correcta*, Bcu – *B. cucurbitae*, Bdo – *B. dorsalis*, Binvadens – *B. invadens*, Bka – *B. kandiensis*, Bole – *B. oleae* and Bzo – *B. zonata*.

## Discussion

Among tephritid fruit flies, morphometric analysis has been tested and successfully used for determining differences among species of the *B. dorsalis* complex and to analyse variability among populations of *Anastrepha fraterculus* (Wiedemann) (Diptera: Tephritidae) collected from different host plants using aculeus, wing and head (frontal plate) characters [25], [28], [29], [45]. Our results showed that *B. invadens* can be morphometrically separated from other *Bactrocera* species (*B. correcta, B. cucurbitae, B. oleae* and *B. zonata*) used in this study with respect to wing morphology and the tibia length. However, the degree of morphological variation with respect to *B. invadens* populations and *B. dorsalis s. s.* used in this study was extremely low (Mahalanobis distance = 11.4). This is in line with the observations of Drew *et al.* [4] who classified *B. invadens* as a member of the *B. dorsalis* complex in possessing a very narrow coastal band and anal streak in addition to other abdominal and thoracic features. In a later study, Drew *et al.* [5] using aedeagus length was able to further discriminate between populations of *B. invadens* from those of *B. carambolae*, *B. dorsalis*, *B. papayae* and *B. philippinensis*. In our study, though the Sri Lankan populations of *B. invadens* were associated with different ecological and biogeographical conditions (i.e. different climate, altitude, vegetational community, etc.) (S.A. Mohamed et al., unpublished data), they showed similar morphology to the African samples, distinct from the other *Bactrocera* species but clustering with the *B. dorsalis s.s.* and supports the fact that the two species are closely related. In another study, Tan *et al.* [22] using chemoecological analysis of phenylpropanoid volatiles in male rectal pheromone gland, showed that males of laboratory-raised *B. invadens* accumulated two metabolites, 2-allyl-4,5-dimethoxyphenol (DMP) and (*E*)-coniferyl alcohol (E-CF) similar to *B. dorsalis* in almost equal quantities, in the rectal sac. On the basis of this finding, the authors concluded that the two pest species are biologically identical. Ongoing mating compatibility studies between *B. invadens* and *B. dorsalis* should further shed more light as to whether the two species belong to the same clade.

In our studies, we also observed that the Mahalanobis distance between *B. invadens* and *B. kandiensis* was short (8.1) indicating that the two species are closely related. Drew *et al.* [4] in the description of *B. invadens* stated that this exotic species in Africa was morphologically similar to *B. kandiensis* based on wing and abdominal characters. The only differentiating characters reported by Drew *et al.* [4] was femora that was entirely fulvous in *B. invadens* and the variation in microtrichia pattern in the basal area of cell br, above cell bm. In a more recent study Drew *et al.* [5] did not find any difference in the thorax length between *B. kandiensis* (3.10 mm) and *B. invadens* (3.10 mm); wing length, *B. kandiensis* (6.18 mm) and *B. invadens* (6.21 mm); wing vein, *B. kandiensis* (2.25 mm) and *B. invadens* (2.25 mm); and hind tibia length, *B. kandiensis* (1.86 mm) and *B. invadens* (1.85 mm). The results of our findings therefore, concur with those of Drew *et al.* [5].

Although morphological characters are primarily used to define species, the genetic and behavioural boundaries of species need to be understood and elucidated. This is particularly true for groups of economically important species such as those in the *B. dorsalis* complex. In this regard, DNA barcoding that involves retrieval of a standard region of mitochondrial gene, Cytochrome c oxidase 1 (CO1) at its 5′ end containing ≈650 base pairs gene (acting as barcode) for identification and delineation of all animal life [39] has shown to be potentially a useful tool to separate members of Tephritid groups of fruit flies [16], [46], [47]. The use of COI sequences together with quantitative support in terms of bootstraps and divergence values provides a better resolution for fruit flies identification than was possible with other methods like the PCR-RFLP [46], [48].

The use of DNA barcodes utilizing the CO1 gene has enabled the interpretation of the relationship between *B. invadens* and the other *Bactrocera* species. In this study, the smallest genetic distance (0.025) was detected between populations of *B. invadens* and *B. dorsalis*, *B. invadens* and *B. kandiensis* (0.057) and *B. dorsalis* and *B. kandiensis* (0.06). This is a typical scenario of divergences between congeneric species which are normally higher than within species [39], [40], [47], [49]. Similar studies by Tan *et al.* [22] using COI gene clustered *B. invadens* and *B. dorsalis* in the same branch. The molecular findings in the current study further substantiate our morphometric data obtained above, in which the Canonical variate plots separated *B. invadens* populations from all the other *Bactrocera* species but matched *B. invadens* populations with *B. dorsalis s.s.* and *B. kandiensis*. This is also evident in the PCA plots using the genetic distances, where *B. invadens* populations clustered with the *B. dorsalis s.s.* and *B. kandiensis*.

Although we recorded some levels of concordance between the molecular and the morphometric results, large divergence at micro-geographic scale was observed among populations of *B. invadens*. For instance, some individuals belonging to *B. invadens* populations of Sri Lanka clustered together with *B. kandiensis* in the NJ trees generated in the study. Therefore, the NJ tree did not fully discriminate the populations belonging to the *B. dorsalis* complex. This is also true in the study by Armstrong and Ball [46] where the COI gene could not confidently separate some of the species within the *B. dorsalis* complex, and hence, have suggested the use of additional gene regions to overcome this limitation.

Some representatives of the *B. dorsalis* complex are extremely polyphagous and highly invasive pests, thus it is one of the most important pest species complexes in world agriculture [1]. Because of their economic and quarantine importance, species-level taxonomic work and diagnostics in the *B. dorsalis* complex is relatively advanced [50], [20], [21], but much effort in this regard is still required. Since the primary goal of DNA barcoding is to develop an accurate, rapid, cost-effective, and universally accessible DNA-based system for species identifications [39], [40], this method could be adopted as a standard method of identification of invasive alien species that pose a high risk on the global economy [46].

The use of DNA barcoding in conjunction with other molecular diagnostic tools, for biosecurity will bridge the limitations of previous molecular tools such as inconsistency in technology use, and finite taxas in some of the invasive species [46]. Several studies have demonstrated the effectiveness of DNA barcoding in different insect groups [39], [40], [51], [52], [46]. These projects have shown that >95% of species possess unique COI barcode sequences; thus species-level identifications are regularly attained [53]. Although DNA barcoding is a current molecular tool of choice and to a certain extent can provide answers to molecular identification (e.g. DNA from incomplete libraries [47]), conclusive phylogeny should include an array of molecular diagnostic tools. Our study has contributed to unraveling the identity of *B. invadens*, a finding which could facilitate its placement in the right phylogeny, potentially easing quarantine restrictions and improvement in the management of this invasive pest. Nevertheless, divergent views still exist. Hence additional studies on the pest chemoecology, behaviour, morphometry and genetics are warranted.

## Materials and Methods

### Ethics Statement

No specific permits were required for the described field studies.

No specific permissions were required for these locations/activities.

The locations are not privately-owned or protected in any way.

The field studies did not involve endangered or protected species.

### Sample collection

Male samples of *B. invadens* were collected (using Methyl eugenol baited traps) from different countries in Africa and South East Asia. The collected insects were preserved in ethanol (70% for morphometric analysis and 95% for DNA analysis). In Africa, the sampling area included regions from the East to West Africa (Data not included). Some representative samples included in the study are from East Africa: from Kenya (Nguruman) and Uganda (Kawanda) and a sample from West Africa, Zaria, Nigeria (Table 5). The South East Asian sample is represented by Sri Lanka (the presumed aboriginal home of *B. invadens*). Also included in this study, were other *Bactrocera* species belonging to the *B. dorsalis* species complex namely *B. dorsalis sensu stricto* (Hendel) (Hawaii) and *B. kandiensis* (Drew & Hancock) (Sri Lanka). Other *Bactrocera* species considered in the study that do not belong to *B. dorsalis* complex were: *B. correcta* (Bezzi) (India), *B. cucurbitae* (Coquillett) (Kenya), *B. oleae* Gmelin (Kenya) and *B. zonata* (Saunders) (Mauritius). The specimens were identified by M.K. Billah (Department of Zoology, University of Ghana, Legon).

**Table 5:** Key of the veins used in morphometrics analysis.

	Representation	Description
vein 1	a_m	Wing length
vein 2	a_b	Humeral break – Subcostal break
vein 3	b_c	Subcostal break – vein R1
vein 4	d_e	r – m
vein 5	e_f	Upper length of dm-cell
vein 6	f_g	Basal height of dm-cell
vein 7	g_h	Lower length of dm-cell
vein 8	h_i	Apical height of dm-cell
vein 9	i_e	Upper length of dm-cell
vein 10	c_j	Vein R1 – Vein R2+3
vein 11	d_k	R_4+5_
vein 12	i_l	M
vein 13	J_k	C
vein 14	n_o	Wing width

### Morphometry

**Figure 6:**
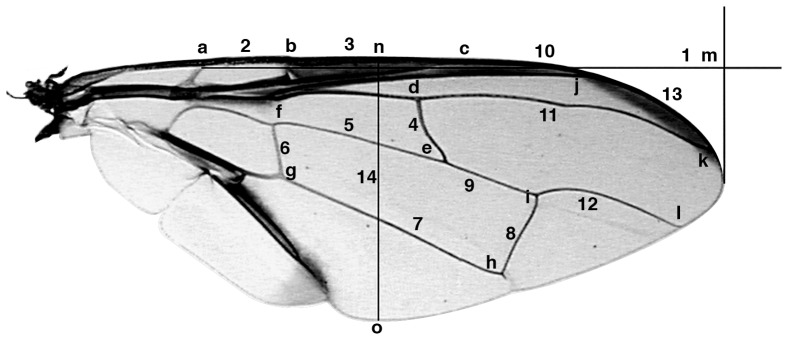
Wing showing points of reading taken for morphometric analysis.

**Figure 7:**
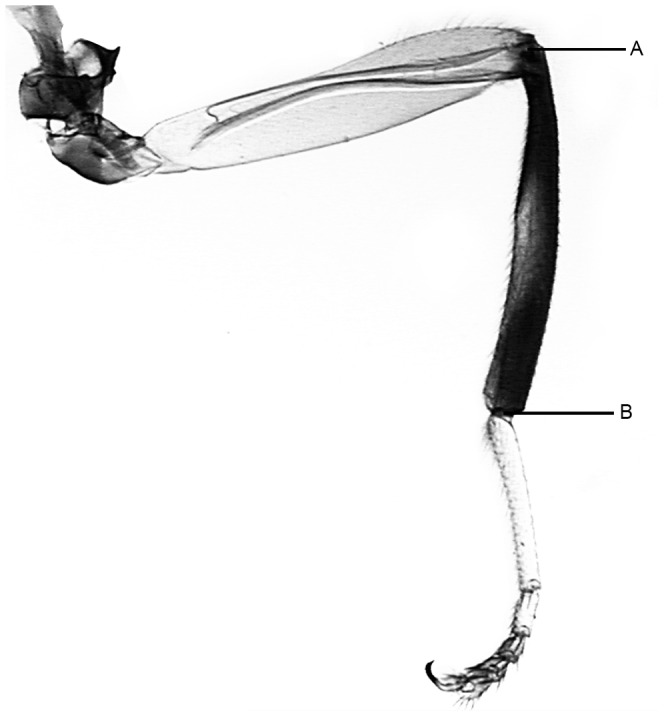
Tibia points of measurement (measurement taken from point A to point B).

Specimens of the different *Bactrocera* species were prepared following the general procedure for slide preparation [54] with modifications according to the needs or state of the specimen. Images of the right wing and right hind tibia from the slide mounted specimens were captured using video microscopy – Leica MZ 125 Microscope, fitted with Toshiba 3CCD camera using the Auto Montage software (Syncroscopy, Synoptics group, 2004) at magnification ×25 for total length and width of the wing, ×50 for the wing veins and ×63 for the tibia. Measurements were taken by the program Image-Pro® Plus version 4.1 for Windows™ (Media Cybernetics, 1999) and the data exported directly to an Excel data sheet. For all parts, measurements were taken in triplicate (to an accuracy of 0.001 mm). Fourteen wing distances between 15 selected landmarks on the wing were computed to characterize the shape and size of the wings for differentiation. These distances are: the Humeral break – Subcostal break, Subcostal break – vein R1, r – m, Upper length of dm-cell, Basal height of dm-cell, Lower length of dm-cell, Apical height of dm-cell, Vein R1 – Vein R2+3, R_4+5_, M, C, the wing length and width, and tibia length (Figure 6 & 7; Table 5). Voucher specimens of all insects and slides are deposited at the Biosystematics Unit of the International Centre of Insect physiology and Ecology (*icipe*), Nairobi, Kenya.

### DNA extraction and PCR

The insects used for DNA barcoding were photographed laterally, dorsally and ventrally and appropriately labeled prior to DNA extraction. DNA from whole insects was extracted using the Qiagen DNeasy® Blood and Tissue Kit (Qiagen, GmbH-Hilden, Germany) as per manufacturer's instructions. The extracted DNA was stored at −20°C until required for amplification. PCR was carried out using universal primers, Forward primer (LCO1490) 5′-GGTCAACAAATCATAAAGATATTGG-3′ and reverse primer (HCO2198) 5′-TAAACTTCAGGGTGACCAAAAAATCA-3′ [55] to amplify a 658 bp fragment of the COI gene. The PCR amplification was carried out in a 20 µl volume containing 1× reaction Buffer, 200 µm of dNTP mix, 0.4 pmol/µl of each primer, 2.5 mM, MgCl_2_, 1 unit *Taq* DNA polymerase (Genescript) and 1 ng DNA template. Standard cycling conditions of 5 min at 94°C, then 35 cycles of 30 s at 94°C, 1 min at annealing temperature of 45°C and 1 min at 72°C, followed by a final elongation step of 5 min at 72°C were used. The products were purified using QIAquick PCR purification kit (Qiagen, GmbH-Hilden, Germany) according to the manufacturer's instructions and subsequently sequenced in both directions using ABI 3700 genetic analyzers. The COI sequences were submitted to the Barcode of Life database (BOLD) and deposited in GenBank (Accession numbers are found in Table 6). The DNA voucher specimens are kept in *icipe* Molecular biology and biotechnology department.

**Table 6:** Collection data of *B. invadens* populations and other *Bactrocera* species used in this study.

Region/Country	Sample name	Sample site	Collection	Coordinates	GenBank Accession numbers
*Bactrocera invadens*					
Africa					
Kenya	Ke	Nguruman	ME	01°48′32S, 036°03′35E	JQ692820, JQ692701, JQ692801, JQ692845, JQ692688, JQ692664, JQ692811, JQ692781, JQ692805, JQ692780
Uganda	Ug	Kawanda	ME	00°49′52S, 031°55′05″E	JQ692633,JQ692709, JQ692824, JQ692854, JQ692650, JQ692794, JQ692844, JQ692752, JQ692681, JQ692841
Nigeria	Nig	Zaria	ME	11°06′N, 07°42′E	JQ692727, JQ692723, JQ692698, JQ692742, JQ692867, JQ692816, JQ692825, JQ692719, JQ692731, JQ692636, JQ692684, JQ692812
Asia					
Sri Lanka	SL	Ranbukpitiya	Tropical almond		JQ692669, JQ692818, JQ692757, JQ692661, JQ692741, JQ692737, JQ692708, JQ692838, JQ692722, JQ692639, JQ692764, JQ692835
*Bactocera correcta*	Bcor	Sri Lanka-Anuradhapura	ME	08°21′0″N, 080°23′1″E	JQ692856,JQ692753, JQ692641, JQ692784, JQ692787
*Bactrocera cucurbitae*	Bcu	Kenya-Nairobi	LT & Cu Lure	01°13′952S, 036°51′314E	JQ692734, JQ692803, JQ692772, JQ692685, JQ692740
*Bactrocera dorsalis s.s*	Bdo	Hawaii	Laboratory reared	-	JQ692775, JQ692829, JQ692694, JQ692790, JQ692747, JQ692706, JQ692864, JQ692758, JQ692678
*Bactrocera kandiensis*	Bka	Sri Lanka-Kandy	ME	07°16′753N, 80°35′731E	JQ692767, JQ692836, JQ692837, JQ692692, JQ692806, JQ692673, JQ692813, JQ692674, JQ692686
*Bactrocera oleae*	Bole	Kenya-Burguret forest	Ex-fruits (olives)	00°06′720S, 37°02′342E	JQ692833, JQ692687, JQ692778, JQ692762, JQ692808
*Bactrocera zonata*	Bzo	Mauritius	Laboratory reared	-	JQ692749, JQ692662, JQ692819, JQ692799, JQ692705

### Data analysis

#### Morphometry

Principal component analysis (PCA), a multivariate statistical procedure commonly used to reveal patterns in measured correlated variables, was used to determine if there was any clustering in the fruit fly species populations based on the wing veins measurements. The data were transformed (log_10_) prior to PCA to stabilize the variance of the measured variables and thus give the variables approximately equal weights in the PCA [56], [57]. Since PCA is inherently a single-group procedure and is not guaranteed to find group differences even if they exist, the log-transformed morphometric data were also subjected to canonical discriminant analysis, a method for analyzing group structure in multivariate data. Bartlett's χ^2^ was used to test for significance of principal components and canonical variates [58], [59]. Mahalanobis squared distances between fruit fly species were obtained as a measure of distance between species based on means, variances and covariances [60]. The analyses were performed using R 2.13.1 [61].

#### COI sequence data analysis

Sequences were assembled and edited using Chromas version 2.13 (Technelysium Pty ltd, Queensland, Australia), and aligned using ClustalX version 1.81 [62]. Phylogenetic and molecular evolutionary analyses were conducted using MEGA version 5 [43] and a Neighbour-joining tree constructed [63] with bootstrapping and using the Kimura 2 distance matrix [42]. A table of between species distances was also constructed using MEGA version 5 [43]. This table of distances was used to generate the principal component plots using GenAlEx 6.41 [44].
